# Impact of Tobacco-Related Health Warning Labels across Socioeconomic, Race and Ethnic Groups: Results from a Randomized Web-Based Experiment

**DOI:** 10.1371/journal.pone.0052206

**Published:** 2013-01-14

**Authors:** Jennifer Cantrell, Donna M. Vallone, James F. Thrasher, Rebekah H. Nagler, Shari P. Feirman, Larry R. Muenz, David Y. He, Kasisomayajula Viswanath

**Affiliations:** 1 Department of Research and Evaluation, Legacy Foundation, Washington, D.C., United States of America; 2 Department of Health Promotion, Education and Behavior, Arnold School of Public Health, University of South Carolina, Columbia, South Carolina, United States of America; 3 Department of Society, Human Development, and Health, Harvard School of Public Health, Center for Community-Based Research, Dana-Farber Cancer Institute, Boston, Massachusetts, United States of America; 4 Larry R. Muenz & Associates, Gaithersburg, Maryland, United States of America; Consejo Superior de Investigaciones Cientifics, Spain

## Abstract

**Background:**

The U.S. Family Smoking Prevention and Tobacco Control Act of 2009 requires updating of the existing text-only health warning labels on tobacco packaging with nine new warning statements accompanied by pictorial images. Survey and experimental research in the U.S. and other countries supports the effectiveness of pictorial health warning labels compared with text-only warnings for informing smokers about the risks of smoking and encouraging cessation. Yet very little research has examined differences in reactions to warning labels by race/ethnicity, education or income despite evidence that population subgroups may differ in their ability to process health information. The purpose of the present study was to evaluate the potential impact of pictorial warning labels compared with text-only labels among U.S. adult smokers from diverse racial/ethnic and socioeconomic subgroups.

**Methods/Findings:**

Participants were adult smokers recruited from two online research panels (n = 3,371) into a web-based experimental study to view either the new pictorial warnings or text-only warnings. Participants viewed the labels and reported their reactions. Adjusted regression models demonstrated significantly stronger reactions for the pictorial condition for each outcome salience (b = 0.62, p<.001); perceived impact (b = 0.44, p<.001); credibility (OR = 1.41, 95% CI = 1.22−1.62), and intention to quit (OR = 1.30, 95% CI = 1.10−1.53). No significant results were found for interactions between condition and race/ethnicity, education, or income. The only exception concerned the intention to quit outcome, where the condition-by-education interaction was nearly significant (p = 0.057).

**Conclusions:**

Findings suggest that the greater impact of the pictorial warning label compared to the text-only warning is consistent across diverse racial/ethnic and socioeconomic populations. Given their great reach, pictorial health warning labels may be one of the few tobacco control policies that have the potential to reduce communication inequalities across groups. Policies that establish strong pictorial warning labels on tobacco packaging may be instrumental in reducing the toll of the tobacco epidemic, particularly within vulnerable communities.

## Introduction

The U.S. Family Smoking Prevention and Tobacco Control Act (FSPTCA) [1] of 2009 required nine new health warning statements to be placed on cigarette packages and in cigarette advertisements by September 2012. The new statements were designed to be accompanied by color images chosen by the Food and Drug Administration (FDA), [2] most of which graphically depict the negative health consequences of smoking. The new warning labels, which are under ongoing litigation as a result of multiple challenges by the tobacco industry, are designed to update the current text-only statements on cigarette packages, which have been in effect in the U.S. since 1984. These text warnings have been consistently characterized as “worn out”–unlikely to be noticed and rated as ineffective by smokers. [3]–[5] Further, studies indicate that the text-only messages had little effect on Americans’ decision-making regarding tobacco use, with the Institute of Medicine describing them as “woefully deficient when evaluated in terms of proper public health criteria.” [4], [6].

Survey and experimental research in the U.S. and other countries supports the effectiveness of pictorial health warning labels (HWLs) compared with text-only HWLs. Data from the International Tobacco Control (ITC) Policy Evaluation Project, a prospective longitudinal panel study of smokers in multiple countries, [7], [8] demonstrate that pictorial HWLs are more likely to be noticed than text-only HWLs, [3], [9]–[11] more effective in informing viewers of the risks of smoking, [10], [12] and more likely to motivate cessation-related activity. [5], [9], [13], [14] Experimental work is limited, but evidence suggests that pictorial HWLs outperform text-only HWLs on a range of outcomes, including capturing attention, [15] increasing awareness of health risks, [16] and creating unfavorable associations with smoking, [17] as well as perceived effectiveness, [18], [19] negative affect, [20] and motivation to quit. [16], [20], [21] However, research on the advantages of pictorial versus text-only warnings has only rarely addressed the issue of differential effects across population subgroups. Research on the effects of pictorial warnings among U.S. ethnic minorities and low SES groups is critical to ensure that this policy addresses, or at least does not exacerbate, tobacco-related health disparities.

A growing body of research has shown that disadvantaged groups may differ in their ability to access, process and act on health information–a phenomenon that has been characterized as communication inequality. [22] This concept expands on components of the knowledge gap hypothesis, which predicts that “as the infusion of mass media information into a social system increases, higher socioeconomic status segments tend to acquire this information faster than lower socioeconomic status population segments so that the gap in knowledge between the two tends to increase rather than decrease.” [23] Studies have documented substantial communication inequalities in access to and processing of information across population subgroups, [22], [24], [25] particularly for lower SES groups. Such inequalities often parallel disparities in smoking-related health knowledge and health outcomes. [26]–[30] Although incompatibility between the level at which information is presented and the audience’s level of literacy and numeracy is typically cited as an important factor, [31], [32] other factors also may influence processing, including differences in the type of messages that attract viewers’ attention, varying interpretation of messages, and variation in the perceived credibility of messages. [33]–[35] Yet the belief that a health message is credible, or the type of message that grabs a smoker’s attention and motivates intentions to quit, may be linked to factors related to an individual’s social class, education or racial/ethnic background.

Evidence on the effectiveness of text and pictorial warning labels by race/ethnicity is sparse. Research in high- and low-income countries examining pictorial labels demonstrates an enhanced effect of the image added to the text consistently across countries, [3], [5], [9], [13], [16], [18], [36], [37] suggesting that the impact of the pictorial HWL may be similarly effective across individuals with different cultural or socioeconomic backgrounds. In a large national focus group study in the U.S., Crawford et al. found that most youth, regardless of race or ethnicity, found the U.S. text-only warnings to be stale and ineffective, with adolescents recommending that future labels be stronger, more direct and even graphic. [38] While not specific to health warning labels, research has found that messages with images of graphic and negative health effects from smoking are often perceived as the most effective among viewers, and this pattern generally holds across racial/ethnic groups. [39], [40] Some research has examined the FDA-approved pictorial labels along with a variety of additional pictorial labels, [41], [42] but these have either not included a text-only condition [18] or have had insufficient sample sizes for fully examining differences in responses by diverse racial/ethnic subgroups. [41], [42] One recent study examining pictorial versus text-only labels, some of which included the FDA-approved images, found no differences by race in cognitive outcomes. [19].

Evidence suggests that pictorial warning labels, particularly those with graphic images, may be more effective among lower SES populations. [11], [18] A cross-sectional comparison of three Latin American countries [11] found that, in the only country with a graphic pictorial HWL, smokers with lower education were more likely than higher educated smokers to think about smoking-related risks and quitting due to HWLs. This evidence is consistent with recent research in the tobacco advertising literature demonstrating that graphic and/or emotionally evocative messages, while associated with strong responses among adults in general, appear to resonate more strongly among lower SES populations.[43]–[46] Research in this area is not conclusive, however, as an experimental study in the U.S. found no differences by education or between white and African American smokers’ in responses to graphic pictorial compared to text only warning labels. [47] Nevertheless, the authors noted that the study may have been underpowered to detect such differences.

Research is needed on cigarette package warning label effects among subpopulations that suffer communication inequalities and tobacco-related health disparities, as interventions to reach these groups have often proven ineffective. The purpose of the present study was to evaluate the potential impact of pictorial warning labels among U.S. adult smokers from diverse racial/ethnic and SES groups. The study was designed to examine differences in cognitive reactions to pictorial labels compared with text-only labels for the new FSPTCA-approved warning messages. The experimental condition included the new pictorial image plus text warning on the front of a cigarette pack in the font size and color mandated by the FSPTCA; the text-only condition was equivalent to the experimental condition but without the pictorial image. Specifically, the study assessed the salience and credibility of the HWLs as well as the perceived impact on smoking behavior and smokers’ intentions to quit. First, we hypothesized that the relative impact of the pictorial label would be higher than the text-only label. Second, given minimal evidence for differences by race/ethnicity, we hypothesized that reactions to pictorial versus text-only warnings would not differ by racial or ethnic status. Lastly, given prior evidence of greater effectiveness of graphic imagery among lower SES populations,[11], [18], [43]–[46] we posited that the greater impact of the pictorial labels, most of which are graphic in nature, compared with the text-only label would be stronger for lower SES groups.

## Methods

### Ethics

The protocol for this study, including online recruitment, informed consent, and data collection, was approved by the Independent Institutional Review Board, Protocol #20036-006.

### Study Design and Recruitment

**Figure 1:**
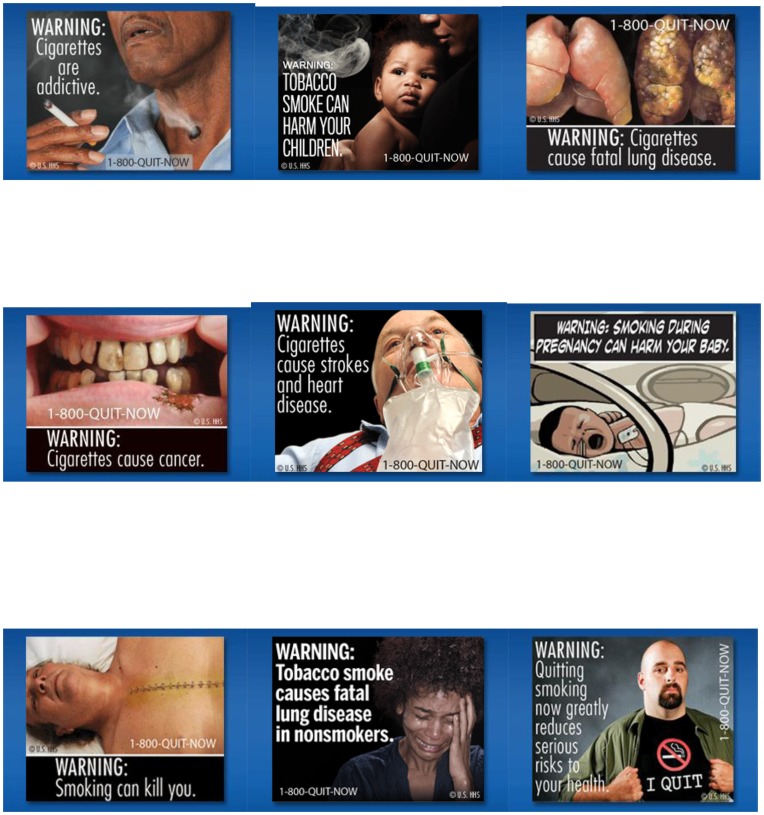
FDA-approved pictorial warning label images. This document includes images of the nine FDA-approved pictorial warning label images used in this study. Reprinted from http://www.fda.gov under a CC BY license, with permission from the FDA, copyright 2012.

A web-based experimental study was conducted with eligible subjects assigned to one of 18 groups. The groups were arranged as a 2×9 factorial with two conditions (text-only and text+ pictorial images) and 9 HWL messages (e.g., “Cigarettes cause cancer”). Participants assigned to the control condition were exposed to one of nine text-only HWLs, and participants in the experimental condition were exposed to one of 9 pictorial HWL with the same text messages as in the control condition. The HWL stimuli included the 9 distinct textual messages and the pictorial imagery designed to accompany them. The stimuli consisted of the front of a plain package of cigarettes, which was approximately 2″ wide by 2.75″ high on the computer screen with the HWL text or HWL text+pictorial covering the front and top 50% of the package. The size, color and font of the text were equivalent in both the text-only and text+pictorial images. The text-only warnings in this study are not equivalent in placement, size or font to the current text-only warnings in the U.S., which are on the sides of packs, in smaller font and in colors that blend in with the color scheme of the pack. The FDA-approved pictorial warning messages can be viewed in Figure 1. After random assignment to one of the 18 groups, study subjects were asked to participate in three waves of data collection; this analysis includes data from Wave 1. Sample size was determined based on a power analysis for examining differences between and within groups over time based on a repeated-measures analysis with between-within interactions, power of 0.95, a moderate effect size of 0.25, an alpha of 0.05.

**Figure 2:**
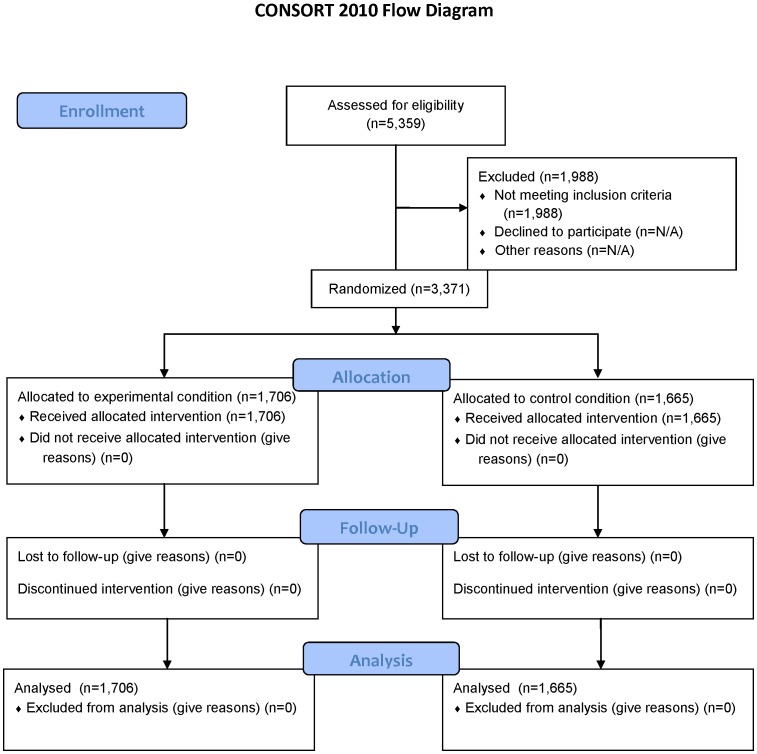
Consort 2010 Flow Diagram. This figure provides information on the study sample for assessment of eligibility (n = 5,359), the number excluded (n = 1,988) and randomized (n = 3,371), as well as the number allocated, followed up and analyzed for the experimental condition (n = 1,706) and control condition (n = 1,665).

The main study sample for Wave 1 consisted of 3,371 adult smokers aged 18 or older who were recruited into the study from a probability-based nationally representative online panel of adults (GfK Group [formerly Knowledge Networks] KnowledgePanel®). Sample participants were also drawn from an online non-probability based opt-in panel from Research Now to provide an additional sample of African American and Hispanic smokers for the study, as these populations were limited in size in the KnowledgePanel®. See Figure 2 for details. Stratified sampling was conducted, and smokers were recruited by three categories of race/ethnicity (white/African American/Hispanic). Potential participants were contacted via e-mail and asked to participate in an online study, with one reminder notice sent. Respondents clicked on a link in their email to access the study’s online consent, which described the study and requested participation. Respondents provided consent when they agreed to participate in the survey. After screening for eligibility, participants were allocated to conditions at Wave 1 based on simple randomization utilizing a SAS random numbers generator. The same randomization procedures, consent process, and questionnaires were used for participants from both panels. For the KnowledgePanel®, the panel recruitment rate was 14.3% and the survey completion rate 50.4%; for the opt-in panel, the survey completion rate for the opt-in panel was 18.0%. [48].

### Sample Characteristics

Of the 3,371 subjects, 1,665 subjects were randomized to the text-only condition and 1,706 subjects were randomized to the pictorial HWL condition. Table 1 provides a profile of sample characteristics. There were no significant differences between participants in the experimental and control conditions with regard to demographic and smoking-related covariates, with the exception of education and readiness to quit: Compared to participants in the experimental condition, those in the control group included slightly more individuals with college education (i.e., 27.8% versus 30.9%,), fewer individuals with some college education (i.e., 39.5% versus 43.6%) and fewer individuals who were ready to quit (21% versus 24.2%). However, these differences were relatively small and only marginally statistically significant. Race/ethnicity, education and income differed across the two panels by design, due to purposive recruiting of specific subgroups of smokers available in each panel. Smoking behaviors also varied between the two panels, with most markers of addiction being somewhat higher among the KnowledgePanel® respondents: for example, KnowledgePanel® subjects smoked significantly more cigarettes per day and had lower readiness to quit compared with the opt-in panel.

**Table 1:** Sample demographics overall and by condition.

	Overall (n = 3,371)	Control (n = 1,665)% or mean (SD1/)	Experimental (n = 1,706)% or mean (SD1/)	p-value (chi-square or t-test)
**Race/ethnicity**				0.434
White	33.4	32.3	34.4	
Black	30.2	30.7	29.7	
Hispanic	36.5	37.0	35.9	
**Income**				0.117
<150% FPL	26.3	24.7	27.7	
150–300%	28.1	28.2	28.0	
>300% FPL	45.6	47.0	44.3	
**Education**				0.045
HS or less	29.1	29.7	28.6	
Some college	41.6	39.5	43.6	
College +	29.3	30.9	27.8	
**Female**	61.2	60.5	61.8	0.451
**Age**	44.17 (14.3)	43.97 (14.1)	44.38 (14.4)	0.404
**Married**	39.9	39.0	40.8	0.294
**Region**				0.914
Northeast	17.5	17.4	17.6	
Midwest	21.5	21.4	21.5	
South	39.0	38.7	39.4	
West	22.0	22.5	21.5	
**Cigarettes per day**	11.76 (9.68)	11.69 (9.91)	11.82 (9.45)	0.700
**Time to smoke**				0.448
<5 minutes	16.4	15.9	16.9	
>5 minutes	83.6	84.1	83.1	
**Ever quit**	78.0	77.9	78.2	0.806
**Readiness – next 30 days**				0.030
No	77.4	79.0	75.8	
Yes	22.6	21.0	24.2	
**Panel Source**				
KnowledgePanel®	52.3	52.2	52.6	0.822
Opt-in panel	47.6	47.8	47.4	

1/ SD = Standard deviation.

### Procedures

Study participants completed the online Wave 1 survey in September 2011, during which they first answered items related to their demographics, smoking behavior and baseline readiness to quit, and then viewed a single image of the front of a generic cigarette pack with either a text-only HWL or pictorial HWL and reported their reactions to the HWL. Participants then answered questions regarding their reactions to the exposure, including cognitive reactions and intention to quit. Participants were able to view the HWL as long as they wanted but were not allowed to go back to the image once they began answering items related to their reactions. The survey took approximately 15–20 minutes.

### Measures

Three outcome measures were developed to capture cognitive reactions related to the salience, perceived impact on smoking behavior, and credibility of the HWL and one outcome measured intention to quit. All were assessed after exposure to the HWL condition. Cognitive reactions were assessed using a 5-point Likert scale of disagreement with a set of statements, where 1 = “not at all” and 5 = “completely.”

Cognitive constructs were based on general principles of communication theory [33], [49], [50] and warning label effectiveness [51]. “Salience” of the message (i.e., whether the message attracts the viewers’ attention, or is attention grabbing) is a critical and distinctive first step in consumer information processing following message exposure [51]. Salience was assessed using two items: the pack is worth remembering and the pack grabbed my attention. Another key dimension of effective warning label communication involves viewers’ judgments on the merits of the warnings – or reasoned beliefs about the consequences of a behavior in light of the information processed. [51] A “perceived impact” construct was designed to capture viewers’ judgments regarding the potential of the warnings to impact their own and others’ smoking behavior and was created from the following three items: the pack makes me want to quit smoking, the pack will make people more concerned about the health risk of smoking, the pack will prevent young people from starting to smoke. For salience and perceived impact, the relevant items were summed and averaged, [18], [52] and internal consistency was assessed (alphas were 0.86 and 0.85, respectively). Yielding to the message is considered a marker of message acceptance in communication theory [53] and is viewed as the point at which attitude change occurs. Yielding cannot occur unless the viewer perceives the message to be credible. A measure for credibility was assessed based on one item: the pack is believable. Responses were dichotomized in the following manner: not at all/a little/some versus a lot/completely. Intention to quit was measured with the question “How likely is it that you will try to quit smoking within the next 30 days?” with response options dichotomized as very unlikely/somewhat unlikely and somewhat likely/very likely.

Covariates included age, gender, race/ethnicity (White/African American/Hispanic), income (<150% federal poverty level [FPL]/150–300% FPL/300%+ FPL), education (high school or less/some college/college or more), marital status (married/other), region of country (Northeast, Midwest, South, West), source of panel (Knowledge Networks/Research Nowpanel), cigarettes per day, time to first cigarette upon waking, any quit attempt, and readiness to quit. Time to first cigarette was dichotomized as within 5 minutes of waking versus more than 5 minutes upon waking. Any quit attempt was assessed by asking whether the participant had ever stopped smoking for 24 hours or longer because they were trying to quit (No/Yes). Readiness to quit was assessed using the following response categories: ready to quit in the next 30 days, the next 6 months or not ready to quit, and was dichotomized as next 30 days vs. next 6 months/not ready.

### Analytic Approach

All conditions were pooled into a single text-only vs. pictorial variable, and regression analyses adjusted for race/ethnicity, education, and income as well as the other covariates described above. We assessed multicollinearity between independent predictors using the Variance Inflation Factor (VIF), as problems with high collinearity can increase standard errors. We used a VIF of 5 as a criterion for excluding variables.[54]–[56] None of the predictors included in the final model had a VIF of greater than 2.5. Next, separate models were run that included the two-way interactions of condition with race/ethnicity, education, and income were included. Outcomes were analyzed using unweighted linear or logistic regression (SAS/STAT software, version 9.2). Primary models for the first hypothesis included an indicator variable for panel source, condition (text-only versus pictorial), demographics and control variables. The second set of models added interaction terms for race/ethnicity by condition and the third and fourth set of models examined interaction of education by condition and income by condition, respectively. Interactions for the nonlinear models were evaluated on both the logit and probability scale and there were no significant differences. [57].

Heterogeneity in the association between experimental condition and the outcomes by source panel were examined. None of these interactions were significant at the p<.05 level and thus were dropped in the final models. Lastly, while the majority of the labels include loss-framed messages and graphic images, there is a striking difference in content and tone in the label that promoted a positive message and image regarding cessation (e.g., “Quitting smoking now greatly reduces risks to your health”) relative to the other warning labels with more graphic images. We therefore ran additional sensitivity analyses with this warning label removed. Results were not significantly different than those based on the full set of warning labels; thus we report on findings from the system of nine warning labels below.

## Results

Table 2 shows the mean scores and percentages for each outcome by key demographic groups for the text-only and pictorial condition. Adjusted regression models examining the pictorial condition compared with the text-only condition (see Table 3) demonstrated significantly stronger reactions for the pictorial condition for each of the outcomes: salience (b = 0.62, p<.001); perceived impact (b = 0.44, p<.001); credibility (OR = 1.41, 95% CI = 1.22−1.62), and intention to quit (OR = 1.30, 95% CI = 1.10−1.53). Overall, Hispanics and African Americans had stronger responses than whites to HWLs, regardless of condition, as did individuals with a high school education or less compared with higher educated individuals for perceived impact and salience. Other predictors of strong reactions overall included ever having quit and readiness to quit. There were no significant differences in reactions across income categories.

**Table 2:** Mean scores or percentages for text-only vs. text+pictorial condition for each outcome by demographic subgroups.

Outcome	Demographic Group		Mean (SD)1/or %		p-value (chi-square or t-test)
Salience	Race/Ethnicity	White	1.94 (1.02)	2.48 (1.18)	<0.0001
		African American	2.39 (1.31)	3.09 (1.36)	<0.0001
		Hispanic	2.29 (1.19)	2.98 (1.32)	<0.0001
	Income	<150% FPL	2.20 (1.22)	2.89 (1.34)	<0.0001
		150–300% FPL	2.23 (1.23)	2.79 (1.27)	<0.0001
		>300% FPL	2.20 (1.17)	2.84 (1.31)	<0.0001
	Education	HS or less	2.18 (1.19)	2.85 (1.29)	<0.0001
		Some college	2.21 (1.21)	2.81 (1.33)	<0.0001
		College or more	2.23 (1.19)	2.88 (1.30)	<0.0001
Perceived Impact	Race/Ethnicity	White	1.91 (0.85)	2.34 (1.05)	<0.0001
		African American	2.34 (1.14)	2.78 (1.18)	<0.0001
		Hispanic	2.23 (1.03)	2.75 (1.22)	<0.0001
	Income	<150% FPL	2.16 (1.03)	2.67 (1.25)	<0.0001
		150–300% FPL	2.16 (1.05)	2.54 (1.15)	<0.0001
		>300% FPL	2.13 (1.02)	2.61 (1.15)	<0.0001
	Education	HS or less	2.17 (1.02)	2.64 (1.19)	<0.0001
		Some college	2.15 (1.04)	2.54 (1.18)	<0.0001
		College or more	2.13 (1.03)	2.66 (1.15)	<0.0001
Credibility	Race/Ethnicity	White	48.4%	53.7%	0.077
		African American	53.7%	63.0%	0.0029
		Hispanic	51.8%	62.5%	0.0002
	Income	<150% FPL	51.2%	58.0%	0.046
		150–300% FPL	50.8%	61.4%	0.0010
		>300% FPL	51.6%	59.5%	0.0021
	Education	HS or less	52.6%	63.9%	0.0004
		Some college	49.7%	56.7%	0.0097
		College or more	52.1%	60.0%	0.013
Intention to Quit	Race/Ethnicity	White	28.9%	35.1%	0.027
		African American	47.9%	54.8%	0.030
		Hispanic	43.7%	49.8%	0.034
	Income	<150% FPL	39.2%	42.4%	0.33
		150–300% FPL	38.1%	45.8%	0.017
		>300% FPL	42.0%	48.8%	0.0078
	Education	HS or less	35.5%	39.0%	0.26
		Some college	38.1%	48.6%	<0.0001
		College or more	47.4%	49.8%	0.46

1/ SD = Standard deviation; FPL = federal poverty level.

**Table 3:** Adjusted regressions for main effects model (experimental condition, race/ethnicity, education, income and covariates) for each outcome.

	Salience		Perceived Impact		Credibility		Intention to Quit	
	N = 3,299		N = 3,303		N = 3,270		N = 3,301	
	Coef.1/	p-value	Coef.1/	p-value	OR^2/^	p-value	OR^2/^	p-value
Text+pictorial**^3/^**	0.63	<.0001	0.44	<.0001	1.41	<.0001	1.30	0.0018
Hispanic**^4/^**	0.33	<.0001	0.34	<.0001	1.32	0.0219	1.42	0.0114
Black**^4/^**	0.42	<.0001	0.39	<.0001	1.38	0.0051	1.79	<.0001
<150% federal poverty level**^5/^**	0.08	0.1728	0.09	0.0796	1.00	0.9673	1.06	0.6159
150–300% federal poverty level **^5/^**	0.06	0.2628	0.05	0.2800	1.06	0.5457	1.04	0.7212
HS or less**^6/^**	0.12	0.0489	0.17	0.0028	1.27	0.0259	0.93	0.5550
Some college**^6/^**	−0.03	0.5636	−0.04	0.4340	0.91	0.302	0.87	0.1619
Knowledge Networks panel**^7/^**	−0.11	0.0675	−0.06	0.2631	0.97	0.7379	0.98	0.8807
Cigarettes per day	−0.003	0.1597	−0.002	0.2988	1.004	0.3871	0.985	0.0026
Time to smoke - <5 min. of waking^8/^	0.04	0.5233	0.02	0.7247	1.001	0.9955	0.75	0.0190
Ever quit - Yes^9/^	0.19	0.0002	0.24	<.0001	0.82	0.0231	2.48	<.0001
Readiness to quit – next 30 days^10/^	0.34	<.0001	0.41	<.0001	0.64	<.0001	12.96	<.0001
Female**^11/^**	0.14	0.0019	0.06	0.1069	0.88	0.0946	1.15	0.1149
18–29**^12/^**	0.08	0.2822	0.01	0.8595	0.94	0.6084	1.29	0.0849
30–44**^12/^**	0.11	0.1054	0.04	0.5487	1.11	0.3629	1.25	0.0949
45–59**^12/^**	0.03	0.6626	0.00	0.9643	1.01	0.9306	1.12	0.3811
Northeast**^13/^**	0.14	0.0357	0.10	0.0826	1.08	0.5335	1.10	0.4744
Midwest**^13/^**	0.11	0.0927	0.03	0.5764	1.07	0.5354	1.05	0.6961
South**^13/^**	0.10	0.0743	0.08	0.1289	1.01	0.9364	1.12	0.3061
Married^14/^	0.03	0.5002	0.02	0.6989	0.87	0.0791	1.11	0.2360

1/ Coef. = Coefficient; **^2/^**OR = odds ratio; **^3/^**Ref = text-only condition; **^4/^**Ref = white; **^5/^**Ref = >300% federal poverty level; **^6/^**Ref = college or more; **^7/^**Ref = Opt-in panel; **^8/^**Ref = time to smoke - >5 minutes of waking; **^9/^**Ref = Ever quit – No; **^10/^**Ref = readiness to quit - >30 days; **^11/^**Ref = Male; **^12/^**Ref = 60 years old or more; **^13/^**Ref = West; **^14/^**Ref = Not married.

**Figure 3:**
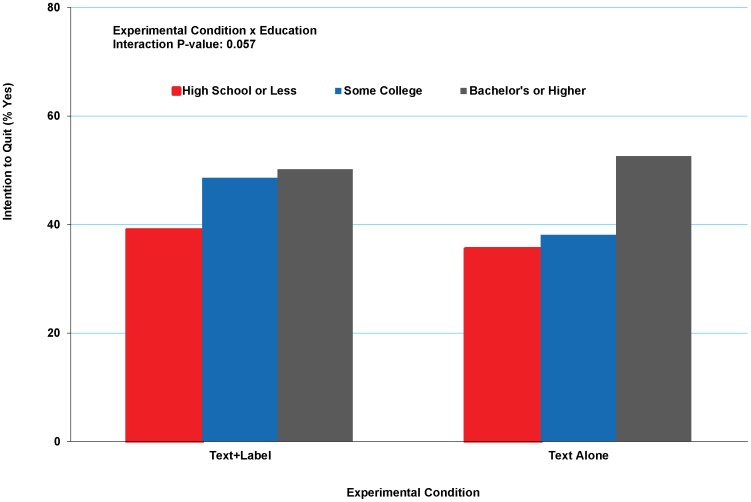
Percentage indicating intention to quit by education level. This figure shows the percentages reporting intention to quit in the next 30 days (yes/no) by exposure to the control or experimental condition for each level of education (high school or less/some college/college or more). In adjusted regression models, the interaction for experimental condition by education was marginally significant at p = .057. The figure indicates a stronger effect for the pictorial condition versus the text-only condition among individuals with moderate education compared with higher educated groups.

No statistically significant results were found examining interactions between condition and race/ethnicity, education, or income, which suggested that the greater impact of the pictorial HWLs compared to the text-only HWL was consistent across these study subpopulations. The only exception concerned the intention to quit outcome, where the condition-by-education interaction was nearly significant (p = 0.057). Figure 3 provides percentages of the intention to quit outcome for each condition by educational categories, indicating a stronger effect for the pictorial condition versus the text-only condition among individuals with moderate education compared with higher educated groups.

## Discussion

The results of this experimental study support and extend prior research regarding the superior performance of pictorial HWLs compared with text-only HWLs on a variety of cognitive outcomes. Findings provide evidence that among racial/ethnic and low-SES subgroups: 1) the new FDA pictorial warnings are, as a whole, more effective than text-only versions of the warnings; and 2) the stronger impact of pictorial warnings is similar across vulnerable population subgroups as compared to text-only warnings. These results were consistent for four separate indicators related to salience, impact, and credibility of the warnings as well their effect on intentions to quit. The latter reaction is particularly important as recent population-based research finds that quit-related cognitions in response to pictorial HWLs is predictive of quitting behavior. [13].

Policy related to warning labels is designed to ensure that consumers are adequately informed of the health risks of smoking and to encourage smokers to consider quitting. However, by only providing factual information, warning labels are often ineffective since consumer information processing is strongly dependent on the presentation and format of messages [58], as well as their ability to stimulate thoughts and emotions. [59] Research on the effectiveness of pictorials is supported by evidence from the fields of advertising, communication and social psychology, which establishes that relevant and vivid pictures are significantly more effective in communicating health and other messages than text alone. [60]–[68] Images designed to illustrate text information stimulate greater message processing because pictures draw attention, improve learning and memory and impact post-message attitudes. [59]–[71] Findings from this study strengthen a growing body of research that demonstrates the greater effectiveness of pictorial HWLs compared with text-only HWLs in conveying risk information, enhancing message processing and stimulating cessation activity. [3], [5], [7]–[11], [13], [15]–[17], [19]–[21] Furthermore, pairing graphic pictorial warnings with plain packaging of cigarettes may increase the effectiveness of health warnings by increasing their salience, recall and credibility [72] and reducing the value smokers place on cigarettes. [47], [73].

Evidence from tobacco countermarketing suggests that graphic messages are some of the most effective message types among all populations. Given that the majority of the FDA warning labels vividly depict the negative health consequences of smoking, their graphic nature may be one factor contributing to similar reactions across groups. [19], [39], [74] Further, cognitive processing may be enhanced by visceral graphic pictures designed to clearly illustrate the meaning of text messages by reducing potential variation across groups in interpretation of textual information due to differences in literacy, culture, language or prior health knowledge. [31], [32], [75], [76] Given disparities in health knowledge, targeted health communications are often called for to respond to the needs of specific groups and reduce potential inequalities in the ability to access, process and act on health information. However, there is a lack of evidence regarding whether targeted communications efforts are more effective than those designed for a general audience in producing desired outcomes and reducing disparities. [33], [77] Results from this study suggest that the FDA-approved pictorial HWLs can achieve their desired effect without exacerbating inequalities, thus enhancing efficiency and cost-effectiveness of warning label policy. [33], [77] Additional research could further examine the effects of each of the nine HWLs separately or systematically compare warning labels, categorized on key dimensions of difference, such as visual content or health topic, to ensure that the individual warning labels or certain types of labels do not have differential effects across diverse groups. However, these warnings are implemented simultaneously as a system, and the critical issue for public health concerns whether they produce differential effects as a whole, when consumers and potential consumers are exposed to HWLs under natural conditions of repeated exposure.

As a population-level intervention, warning labels have the ability to expose smokers, regardless of race/ethnicity or SES, to health messaging on a pack of cigarettes on a consistent basis. Although few differences were found with respect to stimulating higher attention and processing across subgroups, differences in smokers’ ability to act on health information messages can influence health inequalities. [78] Supportive environments, community resources and strong anti-smoking social norms are needed to help reinforce cessation, particularly for lower SES and minority groups who have increased difficulty with cessation. Community mobilization efforts that utilize local institutions to encourage cessation, including the health care system, churches and community-based organizations, can ensure a broader system of support for smokers attempting to quit. [78] Finally, collaborating with community members to buttress social networks and norms that bolster cessation can ensure that the goals of warning label policy are fully realized for all groups. [78], [79].

### Limitations

Results should be considered within the context of the study’s limitations. Since respondents may react differently to warning labels in natural settings, the experimental condition may not perfectly mimic a market environment. The experimental condition draws the attention of respondents toward the package labels, which might be perceived differently when viewed amongst other products in a retail setting or when viewed on a real package, instead of on a computer screen. Experimental research involving HWLs printed on real cigarette packages has produced results that are consistent with those from studies that involve online stimulus presentation, [19], [52] suggesting the validity of our modality of stimulus presentation. Second, we did not have a measure of how many previous smoking-related surveys respondents had taken prior to this study and thus were unable to assess potential conditioning effects, which may reduce the ability to generalize results to the larger population of smokers. Additionally, Asian groups were not represented in this study and all Hispanic respondents spoke English, thus may be more highly acculturated than other Hispanic subpopulations. To ensure sufficient sample sizes of diverse subpopulations, we recruited from two demographically-distinct panels. While there was no evidence overall of a significant condition by panel interaction, the African-American and Hispanic population in the opt-in panel had relatively higher income and education levels, thus possibly limiting generalizability. However, this study was primarily focused on inferences regarding internal validity of the added pictorial image and stronger cognitive processing, which is supported by the consistency of the effect across multiple outcome measures and subgroups. [80] Nonetheless, the use of a web-based opt-in panel to supplement the KnowledgePanel® may have introduced undetected heterogeneity in the experimental effect. Further research is needed to better understand the degree to which combined probability-based and opt-in panels may be utilized to conduct experimental work among disparate populations.

The cognitive outcomes (i.e., salience, perceived impact and credibility) were correlated and show a similar pattern of results. However, we believe that while the correlations found here suggest limitations in the ability to distinctively measure these constructs, the constructs are theoretically distinct and important to consider separately in understanding how consumers process warning label messages and how these reactions may be linked to behavioral intentions and behavior. These constructs are also aligned with those found in other studies examining warning labels, including both ‘real world’ observational studies from the ITC Policy Evaluation project [9], [11], [13], [36] and related experimental work [19], [41], [52]. Further measurement research is warranted to allow for fuller delineation of these constructs in the warning label and related communication literature. Although we did not adjust for multiple comparisons, it is unlikely for false positives to occur because most of the p-values were unambiguously significant and would not have been affected by adjustments. Finally, this study only examines the impact of warning labels at one point in time. Future studies will examine the impact of warning labels with multiple viewings over a period of time.

### Conclusions

Regulatory authorities have the responsibility to ensure that tobacco-related warning messages are conveyed and comprehended by smokers regardless of race/ethnicity or SES. Findings from this study suggest that these pictorial HWLs are similarly effective across key subpopulations. Results are consistent with prior research demonstrating the greater effectiveness of HWLs with vivid pictures for grabbing individuals’ attention and stimulating cognitive reactions that can lead to desired changes in knowledge, attitudes and behaviors around tobacco use. Given their great reach, HWLs may be one of the few tobacco control policies that have the potential to reduce communication inequalities across groups. As most smoking is now among low SES populations, the effectiveness of pictorial HWLs in these groups suggests an opening for effective strategies to communicate risk and promote cessation. Policies that establish strong pictorial HWLs on tobacco packaging may be instrumental in reducing the toll of the tobacco epidemic, particularly within vulnerable communities.
